# Performance Evaluation of Developed Bangasure™ Multiplex rRT-PCR Assay for SARS-CoV-2 Detection in Bangladesh: A Blinded Observational Study at Two Different Sites

**DOI:** 10.3390/diagnostics12112617

**Published:** 2022-10-28

**Authors:** Mamudul Hasan Razu, Zabed Bin Ahmed, Md. Iqbal Hossain, Mohammad Fazle Alam Rabbi, Maksudur Rahman Nayem, Md. Akibul Hassan, Gobindo Kumar Paul, Md. Robin Khan, Md. Moniruzzaman, Pranab Karmaker, Mala Khan

**Affiliations:** 1 Bangladesh Reference Institute for Chemical Measurements, Dhaka 1205, Bangladesh; 2 DNA Solutions Ltd., Dhaka 1207, Bangladesh; 3 Department of Soil, Water and Environment, University of Dhaka, Dhaka 1000, Bangladesh

**Keywords:** Bangasure™, rRT-PCR, SARS-CoV-2, Nucleocapsid, LoD

## Abstract

In this study, we evaluated the performance of the in-house developed rRT-PCR assay for SARS-CoV-2 RNA targeting the envelope (*E*) and nucleocapsid (*N*) genes with internal control as human *RNase P*. A total of 50 positive samples and 50 negative samples of SARS-CoV-2 were tested by a reference kit at site 1 and a subset (30 positives and 16 negatives) of these samples are tested blindly at site 2. The limit of detection (LoD) was calculated by using a replication-deficient complete SARS-CoV-2 genome and known copy numbers, where Pseudo-virus samples were used to evaluate accuracy. On site 1, among the 50 SARS-CoV-2 positive samples 24, 18, and eight samples showed high (Ct < 26), moderate (26 < Ct ≤ 32), and low (32 < Ct ≤ 38) viral load, respectively, whereas in site 2, out of 30 SARS-CoV-2 positive samples, high, moderate, and low viral loads were found in each of the 10 samples. However, SARS-CoV-2 was not detected in the negative sample. So, in-house assays at both sites showed 100% sensitivity and specificity with no difference observed between RT PCR machines. The Ct values of the in-house kit had a very good correlation with the reference kits. LoD was determined as 100 copies/mL. It also displayed 100% accuracy in mutant and wild-type SARS-CoV-2 virus. This Bangasure™ RT-PCR kit shows excellent performance in detecting SARS-CoV-2 viral RNA compared to commercially imported CE-IVD marked FDA authorized kits.

## 1. Introduction

The coronavirus pandemic caused by severe acute respiratory syndrome coronavirus 2 (SARS-CoV-2) is redefining global public health. The disease was first reported in Wuhan, China in December 2019 [1] and since then it has spread throughout the globe. Till now, the world has lost almost 6.2 million lives to this virus. Bangladesh has reported its first COVID-19 case in March 2020. Since then, the number of positive cases has increased at an exponential rate. As of 13 April 2022, the country observed nearly 1.9 million positive cases of COVID-19 with a cumulative death toll of nearly 30,000 [2,3]. Although vaccination started throughout the country, the pandemic might be far away from over. Moreover, the rise of new variants of concerns of SARS-CoV-2 with a higher transmissibility rate is developing a critical challenge to the response strategy. With the continuous threat of contagion, response measures need to evolve. Diagnostic testing is an important pillar of the response measures in this fight against the COVID- 19 pandemic. Clinical symptoms cannot exclusively define COVID-19 diagnosis. Moreover, 40–50% of the confirmed population with COVID-19 are asymptomatic but can easily infect others [4]. Thus, testing will continue to be important for identifying infected individuals and implementing quarantine and treatment measures. It will also become increasingly more important for surveillance and screening efforts to monitor the effectiveness of control measures and carry out informed public health and economic decisions.

Reverse transcriptase-polymerase chain reaction (rRT-PCR) is the gold standard in the detection of SARS-CoV-2. Distinct rRT-PCR testing protocols were swiftly established and made publicly available by the WHO [5] and by the Centre for Disease Control (CDC), USA [6]. To date, Food and Drug Administration (FDA), USA issued over 200 Emergency Use Authorization (EUA) COVID-19 molecular diagnostic kits. However, many of these rRT-PCR kits have a varying range of lower limit of detection (LoD). Therefore, it is necessary to lower the detection limit to ensure the accuracy and reliability of the test results. Many factors might lead to false-negative results, especially low viral loads [7,8,9]. Further, the specificity of the confirmatory test relies on the probe-target sequence. The commercially available rRT-PCR kits generally target nucleocapsid (*N*), envelope (*E*) or RNA- dependent RNA polymerase (*RdRp*) gene of SARS-CoV-2 already published by WHO. However, various mutations have been observed within these regions which might hamper sensitivity [10,11]. Dorp et al. found that about 80% of SARS-CoV-2 genome mutations occur in the spike (S) protein, and a large number of mutations are expressed in the *Orf1ab* [12]. Besides that, Neha Kaushal et. al., studied that no mutational frequency was found at *E*-gene of SARS-CoV-2 genome during the beginning months of the outbreak in the USA [13]. An in-silico study was conducted by Changtai Wang, available from the NCBI and GISAID database, and found that SARS-CoV-2 is relatively conserved, especially in the E, 6, 7b regions where no mutation was found. Hotspot mutations in *ORFs* 1a, S, 8, and the *N* region will cause changes in the amino acid sequences of these proteins, and the effects of these mutations on viral replication, transmission, and the induced immune responses need to be further investigated [14]. Moreover, several types of commercial kits have been developed such as singleplex, duplex, or multiplex. The limitation of the singleplex PCR protocol is the requirement to run three or more PCR reactions per sample because all of the probes are labeled with the same dye. Besides that, singleplex uses large amounts of reagents and reduces the laboratory testing capacity, especially in small-scale facilities, which are crucial during the ongoing COVID-19 pandemic, particularly in developing countries. To improve sensitivity, generally, in commercial kits, multiple probes and primers are used in a multi-step PCR workflow.

Bangladesh, a country in Southeast Asia is a densely populated country with a developing economy. The public health of this country is severely challenged due to the limited number of testing facilities and limited access to locally manufactured rapid diagnostic tests [15]. The country is currently highly dependent on imported test kits which are a major concern for the sustainability of response measures. Globally, there is a scarcity of the resources required for the accurate diagnosis of SARS-CoV-2 and dependence on imported kits develops a critical limiting factor for public health measures mainly due to limited assurance for a continued supply. Further, it is difficult to ensure the high quality and quantity of imported kits. Thus, local manufacturing of high-quality test kits might create assurance of supply with self-reliance for diagnostic testing and offer the potential for price rationalization and expanded access to diagnostics.

In this study, we have developed an in-house multiplex assay against SARS-CoV-2 by targeting two viral gene targets from *E* and *N2* genes named Bangasure™. The primer and probe sequence for SARS-CoV-2 *E* and *N2* gene was previously described by Charité—Universitätsmedizin Berlin Institute of Virology, Berlin, Germany [16] and CDC, USA [17] respectively. The Human *RNase P* gene was included as the internal control [18]. This study determines the performance efficiency of this in-house assay at two sites against two commercially imported CE-FDA approved rRT-PCR kits in determining the SARS-CoV-2 among clinical specimens.

## 2. Methods

### 2.1. Ethical Approval

Bangladesh Reference Institute for Chemical Measurements (BRiCM) in collaboration with DNA Solution Ltd. (DNAS) carried out a subsequent comparative study of Bangasure™ rRT-PCR assay at two sites, DNAS as site-1 and BRiCM as site-2. All procedures in the study were according to ethical standards of the Helsinki Declaration of 1975, as revised in 2010 [19]. Clinical specimens were collected along with case record forms (CRF) of participants were constructed as per Institute of Epidemiology, Diseases Control and Research (IEDCR) from site 1 by DNAS as they have authorization for COVID-19 test by the Government of Bangladesh. This is only a performance evaluation study of Bangasure™ RT-PCR kit in comparison with a CE-FDA marked reference kit by using secondary data and without disclosing or using demographical data of a participant anywhere, so there was no direct subject enrollment, but written consent was obtained from participants during completion of the CRF. Moreover, the study protocol was approved by the institutional ethics review committee (Ref No#BRiCM2206). The Bangasure™ multiplex rRT-PCR efficacy protocol was also accepted and published on the Clinicaltrials.gov site as an NCT05190016 identification number.

### 2.2. Primer and Probes

Primer and probe sequences for SARS-CoV-2 viral target genes previously published by CDC, USA (*N1* and *N2*) [17] and Charité—Universitätsmedizin Berlin Institute of Virology, Berlin, Germany (*E* and *RdRP*) [16] were considered for this study. Through literature review, a multiplex combination of *E*, *N2* along with internal control gene *RNase P* was considered for the study [18]. The primer and probe were ordered from Integrated DNA Technologies-IDT (Coralville, IA, USA). In this article, the *N2* primer and probe will be read as *N* only for future references.

### 2.3. Sample Collection and Preparation

In this study, Oro-pharyngeal swabs from suspected patients were collected in Government approved virus transport medium (VTM) (Sansure Biotech. Inc., Changsha, China) at the outdoor patient department (OPD) of the DNA Solution Ltd. (DNAS), Dhaka, Bangladesh, and transported in a cool box to the laboratory for further processing. RNA extraction was carried out using QIAamp^®^ DSP Virus Spin Kit (Qiagen, Hilden, Germany) according to the instruction manual. Briefly, 200 µL of VTM containing the oropharyngeal swab was employed as starting material for viral RNA extraction using Silica-membrane technology. The samples were lysed, binding to the silica-membrane column, washed to remove contaminants, and eluted with RNase-free elution buffer. Fifty (50) positive and fifty (50) negative SARS-CoV-2 RNA samples were selected by using as reference commercial one-step real-time COVID-19 PCR kit, Novel Coronavirus (2019-nCoV) Nucleic Acid Diagnostic Kit (Sansure Biotech Inc., Changsha, China) following manufacturer’s instruction. The commercial real-time PCR kit uses PCR-Fluorescence probing technology and targets two genes, *ORF 1 ab* and conserved coding regions of the nucleocapsid protein *N* gene by Sansure Biotech kit. Positive internal control of human *RNase P*, along with positive and negative control was used to nullify the presence of PCR inhibitors.

### 2.4. Optimization of Bangasure™ rRT-PCR Assay

Optimization of rRT-PCR reactions of the in-house assay was carried out using four different Real-Time PCR instruments QuantStudio5 (Applied Biosystems, California, USA), BioRad CFX96, and CFX Opus 96 (Bio-Rad Laboratories, Foster City, CA, USA). The in-house assay was optimized via targeting *E* and *N* gene primer/probe published by Charité—Universitätsmedizin Berlin Institute of Virology, Berlin, Germany and CDC, USA respectively along with *RNase P* gene as an internal control [18]. The probes of *E*, *N*, and *RNase P* were labeled with FAM, VIC, and Cy5 to improve multiplexing efficiency. The cycling program was set according to the manufacturer’s instruction of commercial one-step master mix (New England Biolabs, Ipswich, MA, USA). Our optimized protocol consisted of 20 μL reaction mixture containing 5 μL of 4× master mix (New England Biolabs, Ipswich, MA, USA), 2 μL of primers/probes mix, 7 μL extracted RNA or template for positive material (Integrated DNA Technologies-IDT, Coralville, IA, USA), and 6 μL of nuclease-free water (New England Biolabs, Ipswich, MA, USA) with a filter combination of FAM (*E*), VIC (*N*), and Cy5 (*RNase P*).

### 2.5. Limit of Detection (LoD) Determination

To determine LoD, AccuPlex™ SARS-CoV-2 Verification Panel containing replication-deficient recombinant alphaviruses incorporating the full genome of SARS-CoV-2 in known concentrations were ordered from Sera Care (Sera Care Life Sciences, Inc., Milford, MA, USA). The reference materials contained a known concentration of virus particles which were serially diluted starting from 10^5^ Copies/mL to 1 Copy/mL. 5 replications of each dilution series were tested at site 1. These positive materials undergo RNA extraction in the same way as clinical specimens according to the previously described method. The LoD was determined at the lowest concentration at which assay target specific for SARS-CoV-2 was positive for all 5 replicates.

### 2.6. Performance Evaluation of the In-House Assay

Performance evaluation between singleplex and multiplex assay was carried out using synthetic positive control plasmids for *E*, *N*, and *RNase P* gene from IDT. The starting stock for each plasmid control was 2 × 10^5^ copies/μL. They were serially diluted to 2000, 200, 20, and 2 copy copies/μL. Both singleplex and multiplex reactions of our *E*, *N*, and *RNase P* gene-based assay were carried out against these synthetic positive plasmids. To perform clinical evaluation, a total of 100 clinical oropharyngeal specimens were selected containing an equal number of COVID-19 positive and negative samples. The samples (positive = 50 and negative = 50) were analyzed at site 1 using an in-house assay and a commercial multiplex 1copy (1drop Inc., Gyeonggi-do, 13217, Republic of Korea) by a separate analyst and Quant StudioTM 5 real-time PCR detection system (Applied Biosystems, Foster City, CA, USA) where as a subset of those samples (positive = 30 and negative = 16) was reanalyzed using the in-house assay at site 2 by CFX OPUS 96 (Biorad, Hercules, CA, USA). In addition, the efficiency of the in-house one step SARS-CoV-2 real-time PCR assay to detect the SARS-CoV-2 variants of concerns was determined using pseudo virus specimens representing three prominent variants of concerns, i.e., B.1.1.7 (UK variant), B.1.351 (South African variant), and P.1. (Brazilian variant), along with wild type (Wuhan) variant (NC 045512) (Sera Care Life Sciences, Inc., Milford, MA, USA.) and a clinical specimen (OM574617) containing the B.1.1.529 (Omicron variant) of concern.

### 2.7. Accelerated Stability Testing

According to the Arrhenius equation [20], accelerated testing was done to predict stability at both sites independently. The in-house kit which includes Master mix, primers, probes, controls were stored at 4 ± 2 °C both sites and additionally −20 °C for 5 weeks at site 2 which were tested according to previously determined time points. Kits stored at 4 ± 2 °C were used to estimate the shelf life (Table S1) and kits stored at −20 °C were used to evaluate the efficiency of the kit at the actual stored temperature. A panel of specimens positive and negative for COVID-19 was stored as single separate aliquots and analyzed at each time point to determine the in-house assay efficiency. Then results of the kit at 4 ± 2 °C at both sites were compared to the results obtained for the same lot of the kit stored at −20 °C for 5 weeks in site 2.

### 2.8. Data Analysis

Samples were considered positive when the signal detected for *E* and/or *N* genes were detected at Ct < 40. Samples were considered negative when viral target genes had a Ct > 40 or were not detected at all along with the amplified *RP* had Ct < 40. Specimens were labeled as invalid when both *E* and *N* genes along with *RP* signals were undetermined. Nuclease-free water was also used as a no template control (NTC). Data analysis for the commercial kits was performed according to the manufacturer’s instructions. The assay’s sensitivity, specificity, positive predictive value, and negative predictive values were determined using the online version 20.115 of MedCalc statistical software [21].

## 3. Results

### 3.1. Multiplexing of E, N, and RP Genes for Detection of SARS-CoV-2 RNA

Various rRT-PCR reactions with different combinations and concentrations of primer and probes were carried out. Finally, an optimized multiplexing strategy targeting envelops (*E*) and nucleocapsid (*N*) gene of SARS-CoV-2 along with a primer/probe set targeting the human *RNase P* (*RP*) as the internal control was selected. The sequences of the primer/probe considered for the in-house assay are summarized in Table 1.

**Figure 1:**
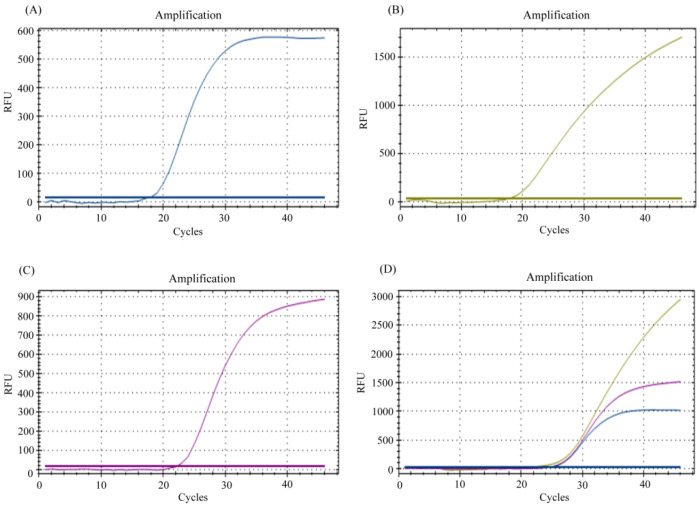
Amplification curves for each of the target genes in singleplex: (**A**) *E* gene, (**B**) *N* gene, (**C**) *RNase P* gene and (**D**) Multiplex assay with the optimized combination of primer-probes.

The optimized combination showed noteworthy amplification for each of the target genes with the commercial one-step master mix (New England Biolabs, Ipswich, MA, USA) in both singleplex and multiplex assay. Ct values from multiplex assay were found to be increased by almost two units when compared with the singleplex assay (Figure 1). An ideal baseline along with the optimum cycle of threshold and minimum background noise was obtained in the following reaction protocol: 25 °C (30 s), 55 °C (10 min), 95 °C (1 min) followed by 45 cycles of 95 °C (10 s), 60 °C (30 s). This optimized protocol and the multiplex assay were compatible in Qunatstudio 5, BioRad CFX96, CFX Opus 96.

### 3.2. Limit of Detection (LoD) Determination of the In-House Multiplex Assay

Replication deficient alphavirus incorporating the whole genome of SARS-CoV-2 was used as reference material to determine LoD. The concentration of starting stock specimen was 10^5^ copies/mL which was serially diluted to 10^4^, 10^3^, 10^2^, 10, and 1 copy/mL. Each dilution series was replicated 5 times and tested against the in-house assay as well as the two commercial rRT-PCR COVID-19 kits considered in this study. The LoD was defined as the lowest concentration at which all replicates (five out of five) were positive for all viral assay targets. The in-house assay showed LoD at 100 copy/mL which is similar to the commercial rRT-PCR kit by Sansure Biotech Inc. However, the other commercial kit by 1drop Inc. (1copy) showed LoD at 1000 copy/mL. The data are summarized in Table 2.

### 3.3. Efficiency of In-House Multiplex rRT-PCR Assay

**Figure 2:**
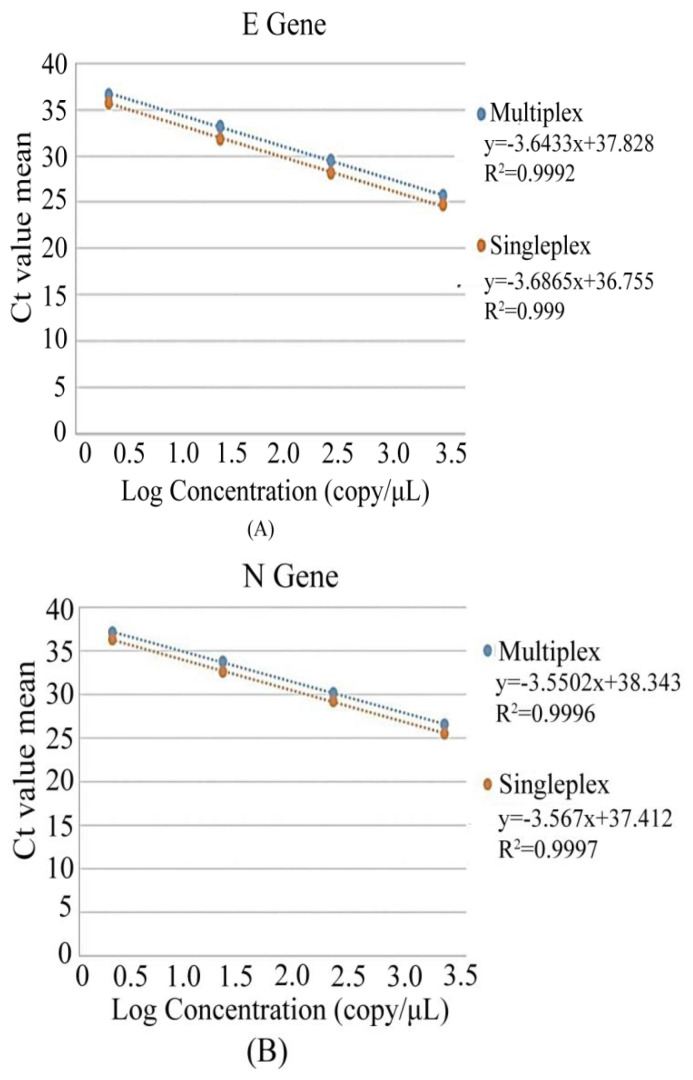

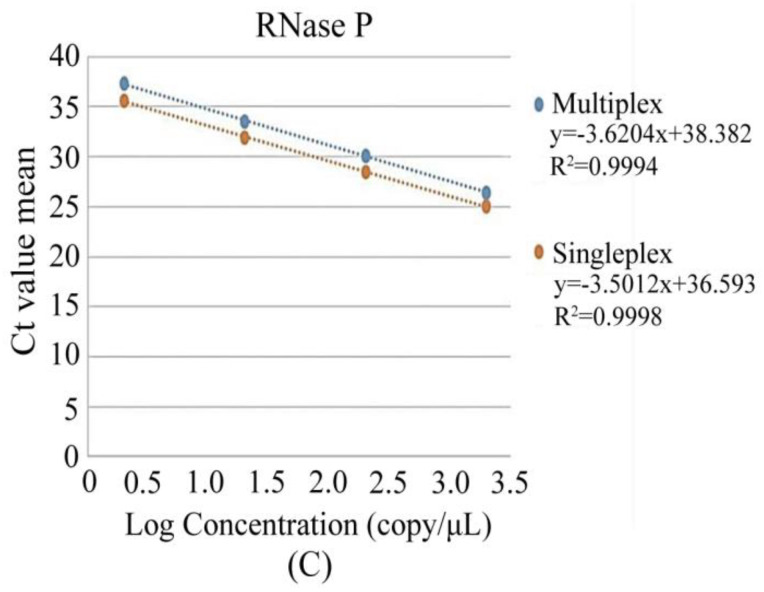
Calculation of calibration curves through singleplex and multiplex rRT-PCR for different copies of positive controls of *E*, *N2* and *RNase P*. (**A**) Standard curve for *E*, (**B**) Standard curve for N and (**C**) Standard curve for *RNase P*.

**Figure 3:**
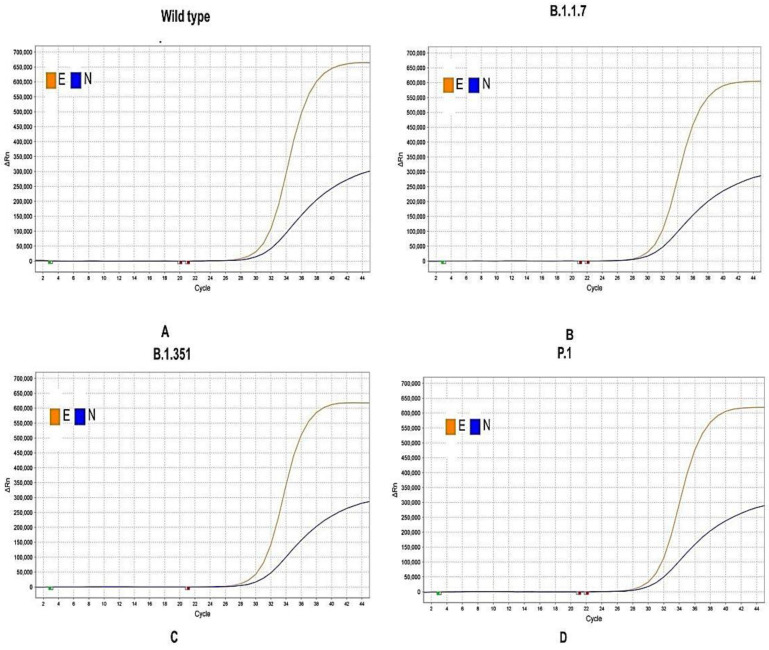

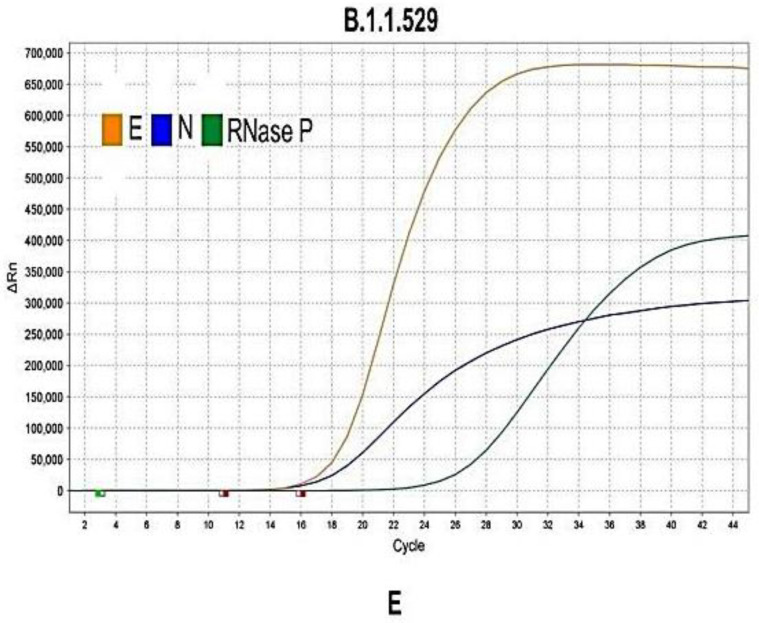
Detection of the SARS-CoV-2 variants of concerns by Bangasure^TM^ multiplex rRT-PCR kit. (**A**) Wuhan variant (wild type), (**B**) UK Variant (B.1.1.7), (**C**) South African Variant (B.1.351), (**D**) Brazilian variant (P.1) and (**E**) Omicron Variant (B.1.1.529) from clinical specimen (OM574617).

Both the singleplex and multiplex reaction of all the assay targets of the in-house assay was carried out against a 10-fold serial dilution of synthetic positive control starting from 2000 copies/μL to 2 copies/μL. The results between singleplex and multiplex showed concordant results with R^2^ > 0.99 (Figure 2). Next, replication-deficient enveloped viruses harboring the mutation of three SARS-CoV-2 variants of concerns (B.1.1.7, B.1.351, and P.1) along with the wild type (Wuhan) variant (NC_045512) were tested with the in-house assay. A clinical specimen (OM574617) containing the variant of concern B.1.1.529 was also tested with the in-house assay. All the viral assay targets were positive against the variants of concerns, including the wild type (Figure 3).

### 3.4. Performance Evaluation of In-House Assay Using Clinical Specimens

**Figure 4:**
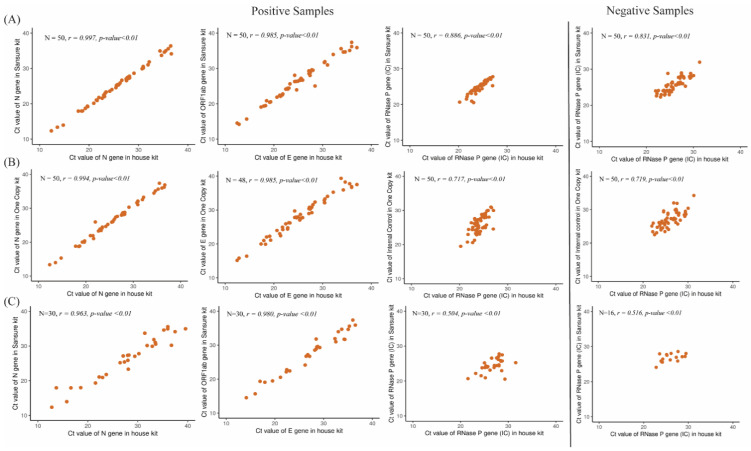
Pearson correlation analysis of Ct values of *E*, *N*, *RNase P* between in house kit and reference kits. (**A**) Pearson correlation between *N*, *RNase P*, *E* gene between In house and Sansure kit at site 1 (Sansure has *ORF1ab* gene instead of *E* gene), (**B**) Pearson correlation between *N*, *RNase P*, *E* gene between In house and One copy kit at site 1, (**C**) Pearson correlation between *N*, *RNase P*, *E* gene between In house and Sansure kit at site 1 (Sansure has *ORF1ab* gene instead of *E* gene).

**Figure 5:**
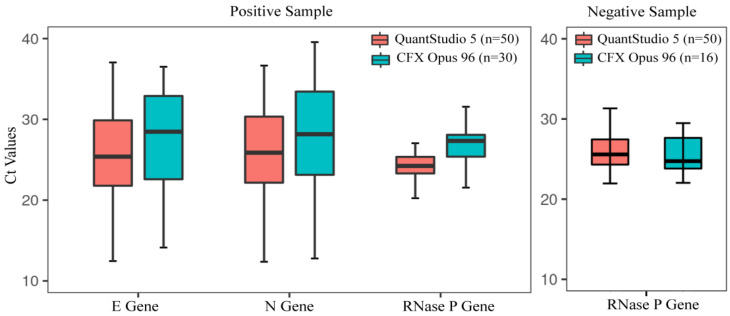
Ct values of individual genes included in in-house kit on two different RT machines used at two sites.

A total of 100 (positive = 50 and negative = 50) clinical specimens (oropharyngeal swab) were considered using the Sansure Biotech PCR kit as a reference kit for the performance evaluation. Among those, at site 1 within the positive samples, 24 samples had high SARS-CoV-2 viral load (Ct < 26) while 18 and eight samples had moderate (26 < Ct ≤ 32) and low viral (32 < Ct ≤ 38) load respectively according to the Sansure COVID-19 rRT-PCR kit. At site 2, within the positive samples, 10 samples had high (Ct < 26), moderate (26 < Ct ≤ 32), and low (32 < Ct ≤ 38) viral load in each group. Besides that, the COVID-19 status of all the samples was blinded and was reanalyzed by the Bangasure™ in-house multiplex kit and 1copy COVID-19 rRT-PCR kits at site 1. The results were then compared with the Sansure data. Both the in-house assays and the 1copy kit accurately identified all the positive and negative samples for COVID-19 at both sites. Thus, in comparison to Sansure kit, the in-house assay has 100% sensitivity, specificity, accuracy, positive prediction value, and negative prediction value (Table 3A,B). The Pearson correlation analysis of *E*, *N*, and *RNase P* gene individually for both positive and negative samples at two sites with the reference kits also indicated a good relationship (Figure 4). Additionally, when compared between the two different RT PCR machines used at two different sites, we found 100% concordance, though the Ct value for each gene at site 2 was found slightly increased (Figure 5).

### 3.5. Determination of Assay Reproducibility and Stability

To determine the in-house assay reproducibility and stability, 10 clinical specimens containing five positive and five negative samples for COVID-19 were used. The specimens were aliquoted and kept at −80 °C to avoid repeated freezing and thawing. The in-house assay was kept in 4 ± 2 °C for the accelerated stability testing in both the sites and additionally −20 ± 5 °C in site 2 to mimic the practical scenario. The samples were tested for five consecutive weeks and each time the tests were replicated five times. Each time, the samples were detected with high precision with minimal deviation from the mean. Further, the coefficients of variation of the precision Ct were less than 5% in site 1 and less than 7% in site 2 at all the storage temperatures for five consecutive weeks. Further from this study, it can be predicted the in-house assay has a stability of at least five months at −20 °C. The data are summarized in Table 4.

## 4. Discussion

Since the beginning of SARS-CoV-2 pandemic, laboratories around the globe faced difficulty to expedite diagnostic tests due to a shortage of resources [22]. This was critical for developing countries that were dependent on imports for diagnostic kits and reagents. Bangladesh, a developing country in south-east Asia, is a densely populated county and was able to cover only 0.66 per 1000 of its citizen under COVID-19 diagnosis [23]. One of the major limiting factors for this poor diagnosis rate is the lack of control over the import supply chain and the quality as well the quantity of the diagnostic kits. As diagnosis is not the most important pillar of COVID-19 pandemic response, it is a critical priority to develop domestically manufactured high volume quality kits. Around the globe, various efforts are ongoing to develop accurate, reliable, and sustainable SARS-CoV-2 detection methods with good sensitivity and specificity [24].

In this study, we have reported the development of a Bangasure™ in-house multiplex rRT-PCR kit for SARS-CoV-2. Two groups of primer-probe sets (Charité/Berlin and CDC designed Primer probe sets) which are recognized globally and used in many diagnostic assays was primarily chosen to be evaluated. Evaluation of these primer probes to find an optimum combination was conducted by thoroughly reviewing the literature [25,26,27,28,29,30]. WHO recommended the Charité/Berlin assay which detects two viral targets *E* and *RdRp* gene [16]. Among these two targets, different studies revealed the sensitivity of the *E* primer-probe is much higher than the *RdRp* primer-probe set [28,30]. Ct values of amplification curves were found to be significantly higher when the *RdRp* primer-probe set was used compared to other recognized primer-probe sets used in the study [30]. On the other hand, comparing the Ct values and analytical sensitivity of *N1* and *N2* primer-probe sets, *N2* performed slightly better than the *N1* primer-probe set [28]. Moreover, the *N1* probe has a single nucleotide mismatch in more than 98% of Omicron variants which is a variant of concern and the most predominant SARS-CoV-2 variant in the world right now [26]. After analyzing all these facts and following the recommendation of Nalla et al., 2020, the *E* primer-probe of Charité/Berlin and *N2* primer-probe designed by CDC was chosen for in-house assay [28].

During the SARS-CoV-2 pandemic, testing demand increased exponentially. Singleplex reactions using various target genes demand more thermal cycler, controls, reagents, and labor which are a limiting factor in resource-poor countries. Studies showed that multiplexing through a single-tube reaction causes a negligible decrease in sensitivity compared to singleplex reaction [31]. Further, mutations are a common possibility of false-negative results. By targeting multiple SARS-CoV-2 viral assay targets, we might have reduced the possibility of false-negative results that might have been raised through any polymorphism within the primer binding site and template region. Thus, the development of a multiplex assay with high sensitivity and specificity will save time, simplify diagnosis, require less reagent, and offer increased test volume which is critical for pandemic response.

In this study, the in-house assay showed great sensitivity and specificity in detecting SARS- Cov-2 efficiently compared to two FDA-approved commercial nucleic acid amplification kits for COVID-19 at both site trials. Though the Ct values for each gene in CFX Opus 96 RT PCR machine at site 2 was found slightly increased when compared with site 1 which used Quantstudio 5 RT PCR machine this might be due to the time gap of analysis between two sites (1 week interval). However, in terms of detection, there was no discordance observed. Analytical sensitivity of the in-house kit was found to be 100 copy/mL, which indicates that samples with low viral loads can be detected efficiently by the in-house kit. The other two commercial RT-PCR kits named Sansure and 1copy showed analytical sensitivity 100 copy/mL and 1000 copy/mL respectively. The nucleic acid extraction method, primer-probe, and other reagents used in reaction might impact the sensitivity of real-time PCR, and thus optimization of an assay is necessary [32]. The linearity (R^2^ > 0.99) we observed in the standard curve and low LoD indicates that the reaction condition used for the in-house assay might have achieved the required optimized condition. Further, all commercial RT-PCR kits along with in-house assay showed amplification in all target genes in both high and low positive samples, which demonstrates the high sensitivity of these kits in a clinical set-up.

In this study, the same set of samples was tested to be compared against all kits, and the initial volume of sample and elution volume were kept concordant for all the samples in the nucleic acid extraction procedure. The extracted RNA was allocated and stored at −80 °C to mitigate the fridge thaw cycle and prevent RNA degradation which might influence the result. These executed procedures enabled us to evaluate the performance of all kits more precisely.

The rise of various variants of concerns has increased the length of this pandemic. These variants contain different mutations in their genome, for example, the “UK Variant” B.1.1.7 contains 23 mutations in *N*, *ORF1ab*, *ORF8*, and S genes [33]. These genetic variations can affect the sensitivity of diagnostic kits by affecting primer binding sites [34]. The in-house assay was tested against different variants to evaluate the efficiency and found it 100% sensitive in detecting different variants, including B.1.1.7, B.1.351, P.1., and B.1.1.529.

As SARS-CoV-2 is a novel virus, the diagnosis is still in a developing phase and different kits show fluctuation in performance. One major aspect of the diagnostic kit is consistency [35]. To evaluate the consistency in a clinical set up, we performed an assay reproducibility and kit stability test where the in-house kit exhibited identical results for five consecutive weeks using a set of ten known samples for evaluation. The kit also showed evidence of stability at 4 °C for the five-week time period in this study, which indicates a sustainable performance of the in-house assay.

The principal feature of our developed in-house assay is that it can be performed in different real-time PCR platforms at different sites. Therefore, this kit can be used in most molecular laboratories for the diagnosis of SARS-CoV-2. Another aspect of the in-house kit is that the master mix used in this assay contains Uracil-DNA glycosylase (UDG) which contributes to eliminating carryover contamination [36]. The primer-probe used in this study is well proven to be used against SARS-CoV-2 virus assay. Hence, we might conclude that the in-house assay will be very specific with minimal chance of a false-positive result.

## 5. Conclusions

We developed an rRT-PCR kit to detect SARS-CoV-2 efficiently compared to kits that are recognized globally. The assay developed in our study can provide a cost-effective solution to support the mass diagnosis of SARS-CoV-2 and reduce the dependency on foreign kits which will make the health care system of Bangladesh more sustainable in during the COVID-19 pandemic.

## Figures and Tables

**Table 1:** Primers and probes used for in-house assay of SARS-CoV-2.

Target Gene	Primer/Probe	Oligonucleotide Sequence (5′–3′)
*E* gene	E_SarbecoF_primer	ACAGGTACGTTAATAGTTAATAGCGT
	E_SarbecoR_Primer	ATATTGCAGCAGTACGCACACA
	Probe_E	FAM-ACACTAGCCATCCTTACTGCGCTTCG-BHQ1
*N* gene	N_cdcF_Primer	TTACAAACATTGGCCGCAAA
	N_cdcFR_Primer	GCGCGACATTCCGAAGAA
	Probe_N	VIC-ACAATTTGCCCCCAGCGCTTCAG-BHQ1
*RNase P*	RP_F_Primer	AGATTTGGACCTGCGAGCG
	RP_R_Primer	GAGCGGCTGTCTCCACAAGT
	Probe_*RNase P*	CY5-TTCTGACCTGAAGGCTCTGCGCG-BHQ-1

**Table 2:** Determining the Limit of Detection (LoD) for the Bangasure^TM^ in-house assay and the two commercial kits using specimens with known copies of replication-deficient alphaviruses incorporating the whole SARS-CoV-2 genome.

Virus Copy/mL	Bangasure^TM^					Sansure Biotec.					1copy						
	Detection Rate, %	Target Gene				Detection Rate, %	Target Gene				Detection Rate, %	Target Gene					
		*E*		*N*			*ORF1ab*		*N*			*E*		*N*		*RdRp*	
		Ct Value		Ct Value			Ct Value		Ct Value			Ct Value		Ct Value		Ct Value	
		Mean	%CV	Mean	%CV		Mean	%CV	Mean	%CV		Mean	%CV	Mean	%CV	Mean	%CV
100,000	100	26.35	2.87	26.83	1.22	100	27.64	0.48	26.46	0.24	100	28.31	0.83	27.77	0.27	28.99	0.34
10,000	100	30.01	0.65	30.21	0.62	100	30.81	0.21	29.56	0.45	100	31.54	0.87	31.34	1.07	32.35	0.81
1000	100	33.52	1.29	34.48	2.40	100	34.77	0.81	33.65	1.97	100	34.99	0.82	34.90	1.63	35.43	0.96
100	100	37.49	1.34	37.71	1.59	100	38.38	1.57	37.11	1.34	0	UND	UND	UND	UND	UND	UND
10	0	UND	UND	UND	UND	0	UND	UND	UND	UND	0	UND	UND	UND	UND	UND	UND
1	0	UND	UND	UND	UND	0	UND	UND	UND	UND	0	UND	UND	UND	UND	UND	UND

UND = undetected.

**Table 3:** Validation and performance determination of the Bangasure^TM^ RT-PCR kit against two commercially available CE-IVD and FDA approved COVID-19 qPCR diagnostic kits (**A**) in site 1 and (**B**) in site 2.

(A)					
Information of Clinical Samples		Number of Samples	Tested by Bangasure^TM^ RT-PCR Kit	Tested by Sansure COVID-19 rRT-PCR Kit	Tested by 1copy 4plex Kit
**Samples Tested positive, *n* = 50**	**High (Ct < 26)**	24	24	24	24
	**Moderate (26 < Ct ≤ 32)**	18	18	18	18
	**Low (32 < Ct ≤ 38)**	8	8	8	8
	**Total**	50	50	50	50
**Samples tested negative, *n* = 50**		50	50	50	50
**Sensitivity,%(95% CI)**			100 (92.89–100)	100 (92.89–100)	100 (92.89–100)
**Specificity,%(95% CI)**			100 (92.89–100)	100 (92.89–100)	100 (92.89–100)
**PPV,%**			100	100	100
**NPV,%**			100	100	100
**Accuracy,% (95% CI)**			100 (96.38–100)	100 (96.38–100)	100 (96.38–100)
**(B)**					
**Information of Clinical Samples**			**Number of Samples**	**Tested by Bangasure^TM^ RT-PCR Kit**	**Tested by Sansure COVID-19 rRT-PCR Kit**
**Sample Tested** **positive, *n* = 30**	**High (Ct < 26)**		10	10	10
	**Moderate (26 < Ct ≤ 32)**		10	10	10
	**Low (32 < Ct ≤ 38)**		10	10	10
	**Total**		30	30	30
**Sample tested negative, *n* = 16**			16	16	16
**Sensitivity, % (95% CI)**				100 (88.4–100)	100 (88.4–100)
**Specificity, % (95% CI)**				100 (79.4–100)	100 (79.4–100)
**PPV, %**				100	100
**NPV, %**				100	100
**Accuracy, % (95%CL)**				100 (92.29–100)	100 (92.29–100)

**Table 4:** Reproducibility of the Bangasure^TM^ multiplex RT-PCR kit in site 1 and site 2 over 5 week.

	Site 1			Site 2					
	Ct Values for Multiplex PCR at 4 °C			Ct Values for Multiplex PCR at 4 °C			Ct Values for Multiplex PCR at −20 °C		
	*E*	*N*	*RNase P*	*E*	*N*	*RNase P*	*E*	*N*	*RNase P*
**Week 1**	21.91	22.75	23.99	23.96	21.52	27.24	25.61	21.52	23.73
**Week 2**	21.44	23.73	24.42	25.068	25.35	25.54	25.35	25.35	25.63
**Week 3**	23.50	23.66	24.76	25.938	24.77	25.94	25.96	24.78	25.84
**Week 4**	22.96	24.35	23.74	26.17	25.13	25.73	26.25	24.89	25.95
**Week 5**	23.44	24.31	23.71	27.256	25.37	26.23	26.89	25.12	25.75
**Mean (SD)**	22.65 (0.93)	23.76 (0.65)	24.12 (0.45)	25.68 (1.24)	24.43 (1.64)	26.14 (0.67)	26.01(0.59)	24.33 (1.58)	25.38 (0.93)
**CV (%)**	4.11	2.73	1.88	4.82	6.73	2.56	2.29	6.52	3.66

## Data Availability

All raw data are provided as Supplementary Materials.

## Supplementary Materials

The following supporting information can be downloaded at: https://www.mdpi.com/article/10.3390/diagnostics12112617/s1.

## Abbreviations

rRT-PCR (real Time Reverse Transcriptase Polymerase Chain Reaction); SARS-CoV-2 (Severe Actute Repiratory Syndrome Corona Virus-2); FDA (Food & Drug Administration); LoD (Lower limit of Detection); DNAS (DNA Solution Ltd.); BRiCM (Bangladesh Reference Institute for Chemical Measurements); Ct (Cycle Threshold); CE-IVD (Certified In Vitro Diagnosis).
