# Supplementary material for: Performance Evaluation of Developed Bangasure™ Multiplex rRT-PCR Assay for SARS-CoV-2 Detection in Bangladesh: A Blinded Observational Study at Two Different Sites
Source: Diagnostics (Basel). 2022 Oct 28;12(11):2617. doi: 10.3390/diagnostics12112617 (PMC9689614; doi:10.3390/diagnostics12112617)
Supplement: Supplementary file 1 [file diagnostics-12-02617-s001.zip › diagnostics-1905964-supplementary.pdf]

Supplementary Table S1: Accelerated stability estimation of the BangasureTM kit in 5-weeks' time period.

If kit is stable at 4±2ºC	Then predicted shelf life at freezer temperature (-20°C±5°C)*
Week 1	1 months
Week 2	2 months
Week3	3 months
Week 4	4 months
Week 5	5 months

*Accelerated stability testing was done according to the following formula derived from the Arrhenius equation; Predicted stability= Accelerated stability x $2^{\Delta T}/10$. Here, ΔT is the difference between the normal storage temperature and the sample storage temperature.

Supplementary Table S2: Raw Ct value of individual gene *at site 1 and site 2*

Site 1

SL. NO.	In house Kit				Sansure				One copy				
	E gene	N gene	Rnasep	Result	N gene	ORF1ab Gene	Rnasep	Result	E gene	N Gene	RdRP	IC	Result
1	22.844	23.422	23.821	Positive	23.319	24.139	24.368	Positive	24.758	24.143	23.787	23.706	Positive
2	30.430	30.996	23.942	Positive	30.145	31.804	24.445	Positive	32.129	32.050	32.036	24.888	Positive
3	25.902	26.282	23.695	Positive	25.871	26.705	23.934	Positive	27.744	27.381	27.245	24.595	Positive
4	21.826	21.993	21.763	Positive	21.777	22.787	22.179	Positive	24.235	25.956	23.312	22.953	Positive
5	30.355	30.963	22.645	Positive	30.553	31.697	23.119	Positive	32.862	32.128	32.179	24.357	Positive
6	30.379	30.952	25.593	Positive	30.215	31.746	25.871	Positive	32.201	31.068	31.992	25.735	Positive
7	12.455	12.368	23.106	Positive	12.337	14.529	20.533	Positive	15.138	13.353	15.322	21.274	Positive
8	14.409	14.780	22.716	Positive	13.955	15.679	20.932	Positive	16.388	15.311	14.358	20.574	Positive
9	23.303	23.470	24.789	Positive	23.326	25.637	25.337	Positive	26.186	24.447	26.105	24.889	Positive
10	21.375	21.631	24.150	Positive	20.927	22.117	24.122	Positive	22.412	21.058	21.744	23.077	Positive
11	21.206	21.499	21.634	Positive	21.070	22.417	21.510	Positive	23.009	21.902	22.853	20.723	Positive
12	19.332	19.673	24.320	Positive	19.325	20.543	23.875	Positive	21.110	19.998	20.685	23.863	Positive

13	18.281	18.696	23.550	Positive	17.984	19.477	22.964	Positive	19.899	18.851	20.261	22.847	Positive
14	17.364	17.830	27.007	Positive	17.927	19.080	25.253	Positive	19.985	18.811	18.747	24.544	Positive
15	30.473	30.752	24.843	Positive	29.924	31.917	24.412	Positive	33.276	31.638	33.377	24.909	Positive
16	22.816	23.224	25.135	Positive	22.915	24.387	25.471	Positive	24.369	23.432	23.963	25.184	Positive
17	25.567	25.750	23.334	Positive	25.404	27.202	23.777	Positive	27.587	26.233	27.118	23.249	Positive
18	27.789	28.180	24.076	Positive	27.803	29.605	24.326	Positive	29.207	28.219	29.086	23.114	Positive
19	25.318	25.556	20.234	Positive	25.189	26.732	20.670	Positive	27.035	25.973	26.376	19.487	Positive
20	27.178	27.511	25.364	Positive	27.040	29.301	25.754	Positive	28.957	27.986	28.749	24.954	Positive
21	32.187	32.182	25.381	Positive	31.858	33.960	26.569	Positive	35.259	33.252	37.595	28.660	Positive
22	22.441	22.971	25.326	Positive	22.445	24.147	26.510	Positive	25.914	23.855	28.240	28.857	Positive
23	25.444	25.995	23.212	Positive	25.361	26.666	24.092	Positive	28.700	26.733	30.745	27.223	Positive
24	24.267	24.634	23.062	Positive	24.021	26.297	24.050	Positive	27.801	25.243	30.073	26.144	Positive
25	20.630	20.960	23.718	Positive	20.149	21.871	24.734	Positive	23.973	21.928	26.105	26.337	Positive
26	17.927	18.506	24.426	Positive	17.941	19.347	25.184	Positive	21.013	18.730	22.857	26.985	Positive
27	18.498	19.256	23.384	Positive	18.700	20.865	24.720	Positive	22.055	20.019	23.885	25.548	Positive
28	21.766	22.659	25.242	Positive	21.569	22.542	25.708	Positive	24.930	23.289	26.575	28.308	Positive
29	19.143	19.733	24.327	Positive	19.202	20.427	25.219	Positive	22.263	20.375	24.375	27.591	Positive
30	12.843	13.592	23.294	Positive	13.370	14.206	24.373	Positive	15.817	13.967	17.562	24.220	Positive
31	27.210	28.199	25.387	Positive	27.317	28.537	26.156	Positive	30.366	28.792	32.659	29.162	Positive
32	27.467	27.889	24.967	Positive	27.138	28.862	25.647	Positive	30.279	28.403	32.359	28.892	Positive
33	24.940	25.690	22.437	Positive	25.226	26.395	23.485	Positive	27.964	26.038	29.720	26.395	Positive
34	27.418	27.578	26.627	Positive	26.559	28.017	27.059	Positive	30.781	28.121	32.200	30.817	Positive
35	24.519	24.202	23.928	Positive	23.568	26.327	24.707	Positive	27.911	24.644	29.888	25.517	Positive
36	27.771	27.910	26.510	Positive	27.388	29.493	27.490	Positive	30.779	28.138	32.519	30.993	Positive
37	23.082	23.130	21.873	Positive	22.070	24.452	22.845	Positive	26.252	23.809	28.633	24.426	Positive
38	35.564	36.525	25.136	Positive	36.370	35.091	25.806	Positive	37.760	36.214	38.455	27.856	Positive
39	34.253	34.361	26.107	Positive	34.957	34.650	27.095	Positive	35.926	34.465	38.349	28.033	Positive
40	31.014	31.939	25.936	Positive	31.030	30.996	26.548	Positive	34.024	32.545	35.682	29.986	Positive

41	33.796	35.249	27.043	Positive	34.618	35.603	27.771	Positive	39.352	35.657	NTD	29.976	Positive
42	35.971	35.513	23.553	Positive	34.980	36.199	24.610	Positive	NTD	37.389	38.884	26.693	Positive
43	37.046	36.036	25.693	Positive	35.579	35.902	26.623	Positive	37.508	35.964	NTD	29.849	Positive
44	35.971	36.655	25.757	Positive	34.144	37.393	26.571	Positive	NTD	36.891	NTD	29.945	Positive
45	24.672	25.443	23.146	Positive	24.562	23.935	23.970	Positive	27.708	25.905	29.465	27.616	Positive
46	34.644	34.662	22.740	Positive	33.701	34.685	23.868	Positive	38.256	35.109	38.675	25.038	Positive
47	27.268	27.750	23.840	Positive	27.402	28.906	24.894	Positive	30.769	28.580	33.331	27.297	Positive
48	28.016	27.869	24.591	Positive	27.531	29.507	25.679	Positive	31.571	28.718	33.874	28.820	Positive
49	28.447	29.095	24.692	Positive	28.645	25.037	25.725	Positive	32.355	30.309	34.685	28.047	Positive
50	24.268	26.642	25.529	Positive	26.665	28.064	26.830	Positive	29.719	27.606	31.62	27.973	Positive
51	NTD	NTD	24.369	Negative	NTD	NTD	24.108	Negative	NTD	NTD	NTD	24.532	Negative
52	NTD	NTD	23.986	Negative	NTD	NTD	23.034	Negative	NTD	NTD	NTD	23.947	Negative
53	NTD	NTD	22.148	Negative	NTD	NTD	22.585	Negative	NTD	NTD	NTD	23.397	Negative
54	NTD	NTD	22.563	Negative	NTD	NTD	24.084	Negative	NTD	NTD	NTD	22.506	Negative
55	NTD	NTD	25.520	Negative	NTD	NTD	26.429	Negative	NTD	NTD	NTD	28.580	Negative
56	NTD	NTD	27.158	Negative	NTD	NTD	27.517	Negative	NTD	NTD	NTD	29.932	Negative
57	NTD	NTD	27.577	Negative	NTD	NTD	28.207	Negative	NTD	NTD	NTD	31.907	Negative
58	NTD	NTD	26.823	Negative	NTD	NTD	27.728	Negative	NTD	NTD	NTD	32.028	Negative
59	NTD	NTD	31.318	Negative	NTD	NTD	31.935	Negative	NTD	NTD	NTD	34.263	Negative
60	NTD	NTD	24.577	Negative	NTD	NTD	25.673	Negative	NTD	NTD	NTD	29.633	Negative
61	NTD	NTD	27.353	Negative	NTD	NTD	28.048	Negative	NTD	NTD	NTD	30.125	Negative
62	NTD	NTD	25.623	Negative	NTD	NTD	24.89	Negative	NTD	NTD	NTD	25.938	Negative
63	NTD	NTD	26.931	Negative	NTD	NTD	25.87	Negative	NTD	NTD	NTD	27.431	Negative
64	NTD	NTD	25.339	Negative	NTD	NTD	25.18	Negative	NTD	NTD	NTD	26.322	Negative
65	NTD	NTD	27.988	Negative	NTD	NTD	27.55	Negative	NTD	NTD	NTD	28.971	Negative
66	NTD	NTD	29.553	Negative	NTD	NTD	27.94	Negative	NTD	NTD	NTD	28.641	Negative
67	NTD	NTD	27.216	Negative	NTD	NTD	26.42	Negative	NTD	NTD	NTD	27.235	Negative
68	NTD	NTD	25.539	Negative	NTD	NTD	26.22	Negative	NTD	NTD	NTD	28.934	Negative

69	NTD	NTD	29.427	Negative	NTD	NTD	27.67	Negative	NTD	NTD	NTD	29.521	Negative
70	NTD	NTD	24.842	Negative	NTD	NTD	23.67	Negative	NTD	NTD	NTD	25.449	Negative
71	NTD	NTD	27.912	Negative	NTD	NTD	27.89	Negative	NTD	NTD	NTD	28.377	Negative
72	NTD	NTD	29.173	Negative	NTD	NTD	27.94	Negative	NTD	NTD	NTD	28.426	Negative
73	NTD	NTD	23.158	Negative	NTD	NTD	22.94	Negative	NTD	NTD	NTD	24.713	Negative
74	NTD	NTD	30.032	Negative	NTD	NTD	28.22	Negative	NTD	NTD	NTD	30.428	Negative
75	NTD	NTD	24.568	Negative	NTD	NTD	28.82	Negative	NTD	NTD	NTD	29.372	Negative
76	NTD	NTD	22.258	Negative	NTD	NTD	23.22	Negative	NTD	NTD	NTD	25.964	Negative
77	NTD	NTD	24.783	Negative	NTD	NTD	23.75	Negative	NTD	NTD	NTD	25.931	Negative
78	NTD	NTD	23.883	Negative	NTD	NTD	22.84	Negative	NTD	NTD	NTD	25.993	Negative
79	NTD	NTD	27.451	Negative	NTD	NTD	28.95	Negative	NTD	NTD	NTD	29.714	Negative
80	NTD	NTD	25.678	Negative	NTD	NTD	24.43	Negative	NTD	NTD	NTD	24.084	Negative
81	NTD	NTD	28.298	Negative	NTD	NTD	25.29	Negative	NTD	NTD	NTD	26.848	Negative
82	NTD	NTD	26.379	Negative	NTD	NTD	25.61	Negative	NTD	NTD	NTD	27.295	Negative
83	NTD	NTD	29.304	Negative	NTD	NTD	27.51	Negative	NTD	NTD	NTD	28.013	Negative
84	NTD	NTD	27.448	Negative	NTD	NTD	28.29	Negative	NTD	NTD	NTD	29.139	Negative
85	NTD	NTD	23.961	Negative	NTD	NTD	23.49	Negative	NTD	NTD	NTD	24.446	Negative
86	NTD	NTD	24.873	Negative	NTD	NTD	22.91	Negative	NTD	NTD	NTD	23.439	Negative
87	NTD	NTD	24.284	Negative	NTD	NTD	25.23	Negative	NTD	NTD	NTD	26.014	Negative
88	NTD	NTD	25.355	Negative	NTD	NTD	26.13	Negative	NTD	NTD	NTD	27.494	Negative
89	NTD	NTD	26.762	Negative	NTD	NTD	26.19	Negative	NTD	NTD	NTD	27.028	Negative
90	NTD	NTD	27.294	Negative	NTD	NTD	25.99	Negative	NTD	NTD	NTD	26.185	Negative
91	NTD	NTD	23.718	Negative	NTD	NTD	24.13	Negative	NTD	NTD	NTD	25.327	Negative
92	NTD	NTD	29.416	Negative	NTD	NTD	28.81	Negative	NTD	NTD	NTD	29.192	Negative
93	NTD	NTD	23.555	Negative	NTD	NTD	24.03	Negative	NTD	NTD	NTD	26.138	Negative
94	NTD	NTD	21.963	Negative	NTD	NTD	23.97	Negative	NTD	NTD	NTD	25.115	Negative
95	NTD	NTD	24.189	Negative	NTD	NTD	25.42	Negative	NTD	NTD	NTD	26.547	Negative
96	NTD	NTD	23.019	Negative	NTD	NTD	22.29	Negative	NTD	NTD	NTD	23.074	Negative

97	NTD	NTD	25.328	Negative	NTD	NTD	23.72	Negative	NTD	NTD	NTD	24.592	Negative
98	NTD	NTD	24.951	Negative	NTD	NTD	24.14	Negative	NTD	NTD	NTD	26.914	Negative
99	NTD	NTD	27.727	Negative	NTD	NTD	25.97	Negative	NTD	NTD	NTD	25.702	Negative
100	NTD	NTD	26.219	Negative	NTD	NTD	25.61	Negative	NTD	NTD	NTD	27.319	Negative

Site 2

Sl No	In house kit				Sansure_DNA lab result			
	N2 gene	E gene	Rnasep gene	Result	N gene	ORF1ab gene	Rnasep gene	Result
1	28.1	26.21	28.06	Positive	23.32	24.14	24.37	Positive
2	31.87	28.47	27.09	Positive	30.15	31.8	24.45	Positive
3	28.02	27.12	25.57	Positive	25.87	26.71	23.93	Positive
4	23.69	22.43	23.17	Positive	21.78	22.79	22.18	Positive
5	33.58	34.36	26.18	Positive	30.55	31.7	23.12	Positive
6	36.74	34.22	28.41	Positive	30.22	31.75	25.87	Positive
7	12.78	14.13	29.3	Positive	12.34	14.53	20.53	Positive
8	15.78	15.95	25.19	Positive	13.96	15.68	20.93	Positive
10	22.95	22.33	25.71	Positive	20.93	22.12	24.12	Positive
11	22.27	23.05	24.31	Positive	21.07	22.42	21.51	Positive
12	21.53	21.14	27.03	Positive	19.33	20.54	23.88	Positive
13	18.58	19.51	28.62	Positive	17.98	19.48	22.96	Positive
14	16.61	17.94	31.56	Positive	17.93	19.08	25.25	Positive
15	32.9	32.33	27.57	Positive	29.92	31.92	24.41	Positive
17	27.33	26.75	24.51	Positive	25.4	27.2	23.78	Positive

18	30.22	28.79	25.32	Positive	27.8	29.61	24.33	Positive
19	26.46	26.41	21.55	Positive	25.19	26.73	20.67	Positive
20	29.4	29.21	28.73	Positive	27.04	29.3	25.75	Positive
21	33.22	33.02	26.51	Positive	31.86	33.96	26.57	Positive
26	13.69	16.95	25.01	Positive	17.94	19.35	25.18	Positive
31	27.98	28.19	27.92	Positive	27.32	28.54	26.16	Positive
32	27.08	28.47	28.47	Positive	27.14	28.86	25.65	Positive
36	28.22	28.61	28.73	Positive	27.39	29.49	27.49	Positive
39	36	35.05	28.01	Positive	34.96	34.65	27.1	Positive
40	33.51	32.51	26.51	Positive	31.03	31	26.55	Positive
41	35.22	35.29	28.09	Positive	34.62	35.6	27.77	Positive
42	39.56	NTD	28.04	Positive	34.98	36.2	24.61	Positive
43	36.02	36.51	27.58	Positive	35.58	35.9	26.62	Positive
44	37.47	36	27.59	Positive	34.14	37.39	26.57	Positive
46	31.41	33.72	25.11	Positive	33.7	34.69	23.87	Positive
51	NTD	NTD	28.62	Negative	NTD	NTD	27.11	Negative
52	38.85	NTD(non-sigmoidal curve)	29.48	Negative	NTD	NTD	28.03	Negative
53	NTD	NTD	27.7	Negative	NTD	NTD	28.59	Negative
54	NTD	NTD	22.8	Negative	NTD	NTD	24.08	Negative
55	NTD	NTD	24.27	Negative	NTD	NTD	26.43	Negative
56	NTD	NTD	25.21	Negative	NTD	NTD	27.52	Negative
57	37.85	NTD(non-sigmoidal curve)	23.95	Negative	NTD	NTD	26.21	Negative
58	NTD	NTD	23.14	Negative	NTD	NTD	27.73	Negative
59	NTD	NTD	23.57	Negative	NTD	NTD	27.94	Negative

60	35.47	NTD(non-sigmoidal curve)	22.03	Negative	NTD	NTD	25.67	Negative
61	NTD	NTD	26.14	Negative	NTD	NTD	28.05	Negative
62	37.87	NTD(non-sigmoidal curve)	27.62	Negative	NTD	NTD	25.89	Negative
63	NTD	NTD	23.9	Negative	NTD	NTD	25.87	Negative
64	33.55	NTD(non-sigmoidal curve)	29.29	Negative	NTD	NTD	27.18	Negative
65	NTD	NTD	24.19	Negative	NTD	NTD	25.55	Negative
66	NTD	NTD	27.31	Negative	NTD	NTD	26.94	Negative

**NTD= Not detected*
